# Association of sarcopenia with mortality and end‐stage renal disease in those with chronic kidney disease: a UK Biobank study

**DOI:** 10.1002/jcsm.12705

**Published:** 2021-05-05

**Authors:** Thomas J. Wilkinson, Joanne Miksza, Thomas Yates, Courtney J. Lightfoot, Luke A. Baker, Emma L. Watson, Francesco Zaccardi, Alice C. Smith

**Affiliations:** ^1^ Leicester Kidney Lifestyle Team, Department of Health Sciences University of Leicester Leicester UK; ^2^ Leicester NIHR Biomedical Research Centre Leicester UK; ^3^ Leicester Real World Evidence Centre University of Leicester Leicester UK; ^4^ Leicester Diabetes Research Centre Leicester UK; ^5^ Department of Cardiovascular Sciences University of Leicester Leicester UK; ^6^ NIHR Applied Research Collaboration (ARC) East Midlands, Diabetes Research Centre Leicester UK

**Keywords:** Sarcopenia, Kidney function, Muscle mass, Strength, UK Biobank

## Abstract

**Background:**

Sarcopenia, a degenerative and generalized skeletal muscle disorder involving the loss of muscle function and mass, is an under‐recognized problem in clinical practice, particularly in chronic kidney disease (CKD). We aimed to investigate the prevalence of sarcopenia in individuals with CKD, its risk factors, and its association with all‐cause mortality and progression to end‐stage renal disease (ESRD).

**Methods:**

UK Biobank participants were grouped according to the presence of CKD (defined as an estimated glomerular filtration rate <60 mL/min/1.73 m^2^) and as having probable (low handgrip strength), confirmed (plus low muscle mass), and severe sarcopenia (plus poor physical performance) based on the 2019 European Working Group of Sarcopenia in Older People and Foundation for the National Institutes of Health criteria. Risk factors were explored using logistic regression analysis. Survival models were applied to estimate risk of mortality and ESRD.

**Results:**

A total of 428 320 participants, of which 8767 individuals with CKD (46% male, aged 62.8 (standard deviation 6.8) years, median estimated glomerular filtration rate 54.5 (interquartile range 49.0–57.7) mL/min/1.72 m^2^) were included. Probable sarcopenia was present in 9.7% of individuals with CKD compared with 5.0% in those without (*P* < 0.001). Sarcopenia was associated with being older; inflammation; poorer renal function; and lower serum albumin, total testosterone, and haemoglobin. The largest risk factors for sarcopenia were having three or more comorbidities (odds ratio: 2.30; 95% confidence interval: 1.62 to 3.29; *P* < 0.001) and physical inactivity: participants in the highest quartile of weekly activity were 43% less likely to have sarcopenia compared to the lowest quartile (odds ratio: 0.57; 0.42 to 0.76; *P* < 0.001). Participants with CKD and sarcopenia had a 33% (7% to 66%; *P* = 0.011) higher hazard of mortality compared with individuals without. Sarcopenic CKD individuals had a 10 year survival probability of 0.85 (0.82 to 0.88) compared with 0.89 (0.88 to 0.30) in those without sarcopenia, an absolute difference of 4%. Those with sarcopenia were twice as likely to develop ESRD (hazard ratio: 1.98; 1.45 to 2.70; *P* < 0.001).

**Conclusions:**

Participants with reduced kidney function are at an increased risk of premature mortality. The presence of sarcopenia increases the risk of mortality and ESRD. Appropriate measurement of sarcopenia should be used to identify at‐risk individuals. Interventions such as physical activity should be encouraged to mitigate sarcopenia.

## Introduction

Sarcopenia describes a generalized degenerative skeletal muscle disorder involving the loss of muscle function and mass.^1^, ^2^, ^3^ In studies of the general population, sarcopenia is associated with increased falls, functional decline, frailty, and premature mortality.^1^, ^4^, ^5^ Sarcopenia can double individual healthcare costs and the annual cost of low muscle mass in the USA is estimated to total >$18.5 billion.^6^

Owing to large discrepancies in definitions,^7^ sarcopenia remains a somewhat under‐recognized problem with only half of geriatric healthcare professionals undertaking any form of sarcopenia assessment.^8^ As well as forming part of the definition of ‘protein‐energy wasting’ in nephrology, sarcopenia is confounded by terms such as ‘malnutrition’ and ‘cachexia’, confusing clinicians and reducing its clinical utility.^9^ Possibly, the most widely accepted definition of sarcopenia is that from the European Working Group of Sarcopenia in Older People (EWGSOP), which recently promoted weakness, above low muscle mass, as its primary indicator.^4^ Whilst sarcopenia can be ‘confirmed’ and graded for severity with further assessments of muscle mass and physical performance, the concept of ‘probable sarcopenia’ (defined as low muscle strength) is intended for better practical recognition and management of sarcopenia.

Chronic kidney disease (CKD) is described as a model of ‘accelerated ageing’ due to the characteristic aberrant changes in muscle mass and function observed.^9^ In CKD, sarcopenia pathophysiology is complex and, although partly driven by ageing,^10^, ^11^ may be exacerbated by the accumulation of uremic toxins, inflammation, insulin resistance, malnutrition, oxidative stress, ubiquitination, and physical inactivity.^1^, ^9^, ^11^, ^12^, ^13^ In patients with end‐stage renal disease (ESRD) and on dialysis, sarcopenia has a prevalence of between 20% and 55%^14^, ^15^, ^16^, ^17^, ^18^ and is associated with an increased mortality risk.^13^, ^14^, ^16^, ^18^, ^19^ However, the prevalence in earlier non‐dialysis CKD stages is less well‐defined, and the association with unfavourable outcomes is poorly understood.^9^, ^12^, ^20^, ^21^ Given that sarcopenia characteristics (e.g. low muscle function) may be ameliorated through appropriate interventions, such as exercise, there remains an important opportunity to address sarcopenia in the earlier CKD stages.

Previous studies of sarcopenia in non‐dialysis CKD patients are limited by low sample sizes, lack of control group, and inconsistent use of definitions^12^, ^21^, ^22^, ^23^ The aims of this study were to (i) identify the prevalence of sarcopenia in a large cohort of individuals with CKD; (ii) identify the risk factors of sarcopenia in those with CKD; and (iii) explore the association of sarcopenia with all‐cause mortality and progression to ESRD.

## Materials and methods

### Data source and study population

This study was conducted following the STROBE guidelines for reporting observational studies (STROBE checklist is reported Data S1). The UK Biobank is a large prospective epidemiological study designed to investigate the role of genetic, lifestyle, and environmental factors in health and disease.^24^ In summary, extensive data on a range of demographic, clinical, lifestyle, and social outcomes were collected from 502 536 participants aged between 37 and 73 years across the UK between 2006 and 2010. The UK Biobank was approved by the North West Research Ethics Committee (06/MRE08/65).

### Definition of chronic kidney disease and comorbidities

Serum creatinine values were used to estimate glomerular filtration rate (GFR) using the ‘Chronic Kidney Disease Epidemiology Collaboration’ (CKD‐EPI) formula^25^; participants without a creatinine value were excluded. CKD was defined as an estimated GFR (eGFR) <60 mL/min/1.73 m^2^, based on the assessment of creatinine during the UK Biobank baseline visit. Mild to moderate CKD is defined as an eGFR of between 59 and 30 mL/min/1.73 m^2^.^25^ Individuals with eGFR ≥60 mL/min/1.73 m^2^ constituted the non‐CKD group.

Comorbidities were defined among the chronic conditions identified by Chudasama *et al*.^26^ excluding CKD. We then identified if each participant had one or more codes relating to each chronic condition and scored one point for each chronic condition identified (i.e. 0, 1, 2, or 3 or more comorbidities). All respondents with cancer were excluded a priori due to large confounding effects on cachexia. Information on prevalent cancer and non‐cancer conditions were self‐reported. A full list of comorbidities can be found in Data S2.

### Definition of sarcopenia status

As per the EWGSOP criteria,^4^ ‘probable sarcopenia’ was defined as a maximum handgrip strength (HGS) < 27 kg in male and <16 kg in female patients, measured using Jamar J00105 hydraulic handheld dynamometer. One measurement was taken in each hand with the participant seated upright and their forearms placed on armrests. The maximum value, from either hand, was recorded.

Participants were diagnosed with ‘confirmed sarcopenia’ if they had both low HGS *and* low muscle mass. Muscle mass was assessed using bioelectrical impedance analysis (Tanita BC 418MA Body Fat Analyser). Individuals unable to undergo this assessment included those who were pregnant, using a pacemaker, and those unable to stand. Appendicular fat‐free mass (summed values of the arms and legs) was transformed into appendicular lean mass (ALM).^27^ ALM was expressed relative to height (m^2^) using standing height measured by a SECA 2020 height measure. Low muscle mass was defined as ALM/height^2^ < 7.0 kg/m^2^ in male and <5.5 kg/m^2^ in female patients.^4^ Given the interaction of body mass with sarcopenia and limitations of ALM normalized for height^2^,^28^ we also adjusted ALM for body mass index (BMI) (ALM^BMI^) using the criteria proposed by the Foundation for the National Institutes of Health Sarcopenia Project (FNIHSP), with a low muscle mass defined as ALM^BMI^ < 0.789 in male and <0.512 in female patients.^28^

The UK Biobank does not contain an objective measure of gait speed as specified by the EWGSOP and FNIHSP criteria. As a surrogate marker of poor gait speed and low performance, we considered participants who self‐reported being unable to walk or their walking pace as ‘slow’. Slow self‐reported walking speed is an established marker of low physical performance in older adults,^29^ and walking speed has been used to define low performance in sarcopenia assessment.^27^ Participants who satisfied *all* criteria (i.e. low strength, low muscle mass, and poor performance) were diagnosed with ‘severe sarcopenia’. Further details on these procedures can be found in Data S3.

### Other risk factors

Serum C‐reactive protein (CRP), cystatin C, albumin, haemoglobin, vitamin D, testosterone, IGF‐1, and HbA1c were included among the possible confounders. Multiple immunoassays and clinical chemistry analysers were used to measure these biochemistry markers (Data S4). Other variables included the number of meat and fish servings per week and physical activity levels. As a surrogate measure of protein intake, the number of oily fish, non‐oily fish, processed meat, poultry, beef, lamb, and pork intake servings per week were taken from a touchscreen questionnaire completed during the assessment visit. This questionnaire is a valid indicator of dietary intake.^30^ Self‐reported physical activity was measured using the short form International Physical Activity Questionnaire and metabolic equivalent of task (METs) from exercise were calculated using the fields relating to frequency and duration of pleasure walking, strenuous sport, and other exercises (Data S5).

### Outcomes

All‐cause mortality was determined from data linkage to national death Registries (NHS Information Centre for participants from England and Wales and NHS Central Register, Scotland, for participants from Scotland). ‘Incident ESRD’ was defined at the point at which patients began treated with renal replacement therapy (RRT). ESRD was identified in hospital admissions data using ICD‐10 and OPCS4 codes. Participants who received a kidney transplant or peritoneal dialysis were assumed to be ESRD cases. To exclude cases of AKI, the remaining RRT cases were deemed to be ESRD cases only if they have an associated indicator of CKD Stage 5 in the 365 days preceding identification of having ESRD. This algorithm has previously been used to successfully identify those with ESRD in the UK Biobank.^31^ Participants identified with existing ESRD at baseline visit (either detected by hospital admission records or self‐report) (‘prevalent ESRD’) were excluded from the analysis. Date of death and date of ESRD report were censored at the date of last update in Biobank, which were respectively 15 February 2018 and 19 January 2018. Further details can be found in Data S6 and S7.

### Statistical analysis

Summary measures were described using mean (standard deviation) or median (interquartile range) for continuous variables and as a count (percentage) for categorical ones. Means were compared using a two‐sample *t* test and medians with a two‐sample Wilcoxon test. Count data were compared using a *χ* ^2^ test.

**Figure 1:**
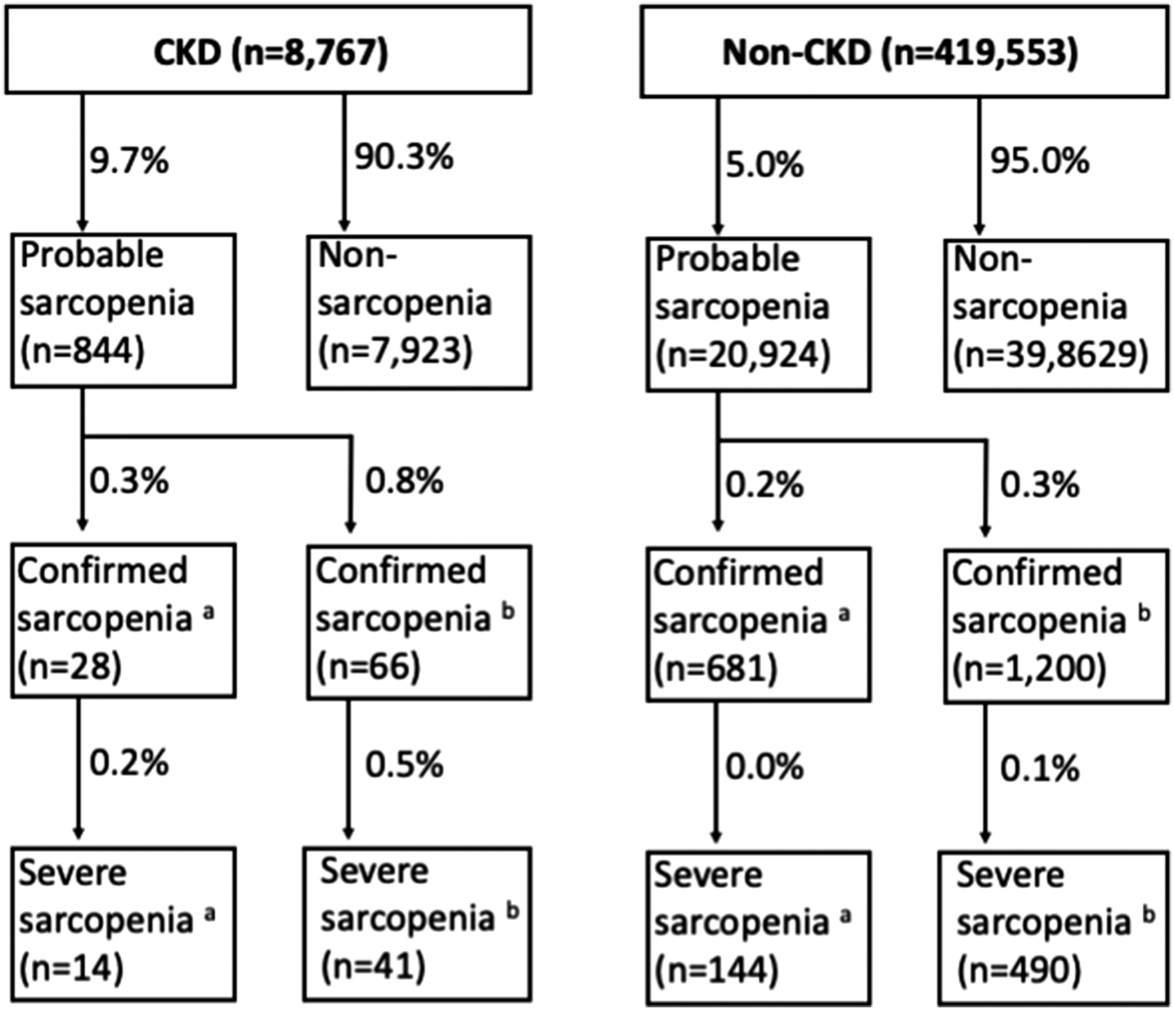
Prevalence of sarcopenia status in chronic kidney disease (CKD) and non‐CKD participants. ^a^Low muscle mass defined as per European Working Group of Sarcopenia in Older People (appendicular lean mass adjusted for height2); ^b^low muscle mass defined as per​ Foundation for the National Institutes of Health Sarcopenia Project (FNIHSP​) (appendicular lean mass adjusted for body mass index). Missing data for these variables can be found in Table S9.

Statistical modelling was restricted to probable sarcopenia to ensure an adequate case number (*Figure* 1). Cross‐sectional associations between potential risk factors and probable sarcopenia were explored using logistic regression analysis. Age, sex, ethnicity, height, weight, number of comorbidities, CRP, albumin, BMI, cystatin C, total testosterone, vitamin D, IGF‐1, HbA1c, haemoglobin concentration, METs from exercise per week, and number of meat servings per week were considered potential risk factors. The modelling assumption of linearity to the log odds was tested by producing a scatter plot of each continuous variable against the log odds and checking for linearity, due to failing this assumption CRP, and cystatin C were categorized at the median value and METs from exercise were categorized into quartiles for analysis. Participants with missing data on any of the variables included were excluded from these models. Missing data can be found in *Tables* S8–S10.

Associations between probable sarcopenia and mortality were reported as hazard ratios estimated using flexible parametric survival models, with time into the study (baseline visit to event/censoring) as time scale.^32^ Unadjusted and adjusted models were fitted with significant (*P* < 0.050) risk factors used as potential confounders, grouped as non‐modifiable (Model 1: age, ethnicity, sex, and number of comorbidities) and modifiable (Model 2: CRP, albumin, BMI, testosterone, and haemoglobin concentration). This model was also used to calculate standardized survivals at 10 years and their difference. An adjusted model was also fitted with the CKD and non‐CKD groups both included in the model, with CKD status included as a variable, with an interaction between probable sarcopenia and CKD exposure fitted to investigate whether the association of probable sarcopenia with morality is modified by CKD status. In those with CKD, the same modelling approach was used to estimate hazard ratios for ESRD in unadjusted and adjusted models, which included age, sex, ethnicity, and number of comorbidities being identified a priori as potential confounders. Analyses were conducted using Stata 16 (StataCorp) and R (4.0.2), and results are reported with 95% confidence interval (CI); a *P* value <0.05 was deemed statistically significant.

## Results

### Participant characteristics

Among the 428 320 participants included in the study, 8767 (2%) had CKD (*Table* 1). Participants with CKD presented with mild to moderate disease: the majority (85%) was in Stage 3a and the median eGFR was 54.5 mL/min/1.72 m^2^. Compared with non‐CKD participants, those with CKD were older, had more comorbidities, higher CRP, lower haemoglobin, higher testosterone, and higher HbA1c. Those in the CKD group had a higher BMI, body fat %, absolute ALM, and ALM/height^2^; however, ALM^BMI^ was reduced. Participants with CKD had poorer HGS and slower walking speed. There were no differences in sex, and the majority (~95%) of participants was of White ethnicity. Participant characteristics stratified by sarcopenic status can be found in *Table* S11.

**Table 1:** Participant characteristics

	CKD (*n* = 8767)	Non‐CKD (*n* = 419 553)	*P* value
Age, years	62.8 (5.8)	56.1 (8.1)	<0.001
Sex, male, *n* (%)	4055 (46%)	195 570 (47%)	0.510
Ethnicity			
White	8346 (95%)	396 213 (94%)	0.002
Other	421 (5%)	23 340 (6%)	
No. of comorbidities			
0	1977 (23%)	195 716 (47%)	<0.001
1	2942 (34%)	139 696 (33%)	
2	2168 (25%)	58 102 (14%)	
≥3	1680 (19%)	26 039 (6%)	
Albumin, g/L	44.4 (2.9)	45.3 (2.6)	<0.001
eGFR, mL/min/1.72 m^2a^	54.5 (49.0–57.7)	93.3 (84.1–100.5)	<0.001
Stage 3a, *n* (%)	7423 (85%)	—	—
Stage 3b, *n* (%)	1079 (12%)	—	—
Stage 4, *n* (%)	238 (3%)	—	—
CRP, mg/L^a^	1.98 (0.99–4.12)	1.30 (0.64–2.70)	<0.001
Haemoglobin, g/dL	13.8 (1.4)	14.2 (1.2)	<0.001
Testosterone, nmol/L^a^	6.0 (1.0–10.8)	5.58 (1.0–11.7)	<0.001
HbA1c, mmol/L	39.3 (9.2)	36.0 (6.7)	<0.001
Body mass, kg	82.1 (16.4)	78.1 (15.9)	<0.001
BMI, kg/m^2^	29.3 (5.2)	27.4 (4.7)	<0.001
Body fat, %			
Males	27.2 (5.8)	25.2 (5.8)	<0.001
Females	39.1 (6.8)	36.5 (7.0)	<0.001
ALM, kg			
Males	27.2 (4.2)	27.0 (3.9)	0.013
Females	19.0 (2.7)	18.4 (2.4)	<0.001
ALM/height^2^			
Males	8.9 (1.2)	8.7 (1.1)	<0.001
Females	7.3 (1.0)	7.0 (0.8)	<0.001
ALM^BMI^			
Males	0.93 (0.1)	0.98 (0.1)	<0.001
Females	0.66 (0.1)	0.69 (0.1)	<0.001
Handgrip strength, kg			
Males	38.8 (9.3)	41.9 (9.0)	<0.001
Females	23.4 (6.6)	25.2 (6.4)	<0.001
Slow walking speed, *n* (%)			
Males	842 (21%)	14 290 (7%)	<0.001
Females	882 (19%)	17 058 (8%)	<0.001

BMI, body mass index; ALM, appendicular lean mass; CKD, chronic kidney disease; CRP, C‐reactive protein; eGFR, estimated glomerular filtration rate.

^a^ Median and interquartile range.

Data presented as mean and standard deviation, unless otherwise indicated.

Missing data for these variables can be found in Data S7.

### Prevalence of sarcopenia

The unadjusted prevalence of sarcopenia was approximately double in participants with CKD compared with those without, regardless of definition used (*Figure* 1). In participants with CKD, 9.7% had probable sarcopenia, twice the prevalence in those without CKD with 5.0% (RR: 1.93 *P* < 0.001). Using the EWGSOP and FNIHSP criteria, respectively, 0.3–0.8% of the CKD group had confirmed sarcopenia and 0.2% had severe sarcopenia. In contrast, 0.2–0.3% and 0.0–0.1% of those without CKD had confirmed and severe sarcopenia, respectively (all *P* < 0.001).

### Risk factors for sarcopenia in those with CKD

**Figure 2:**
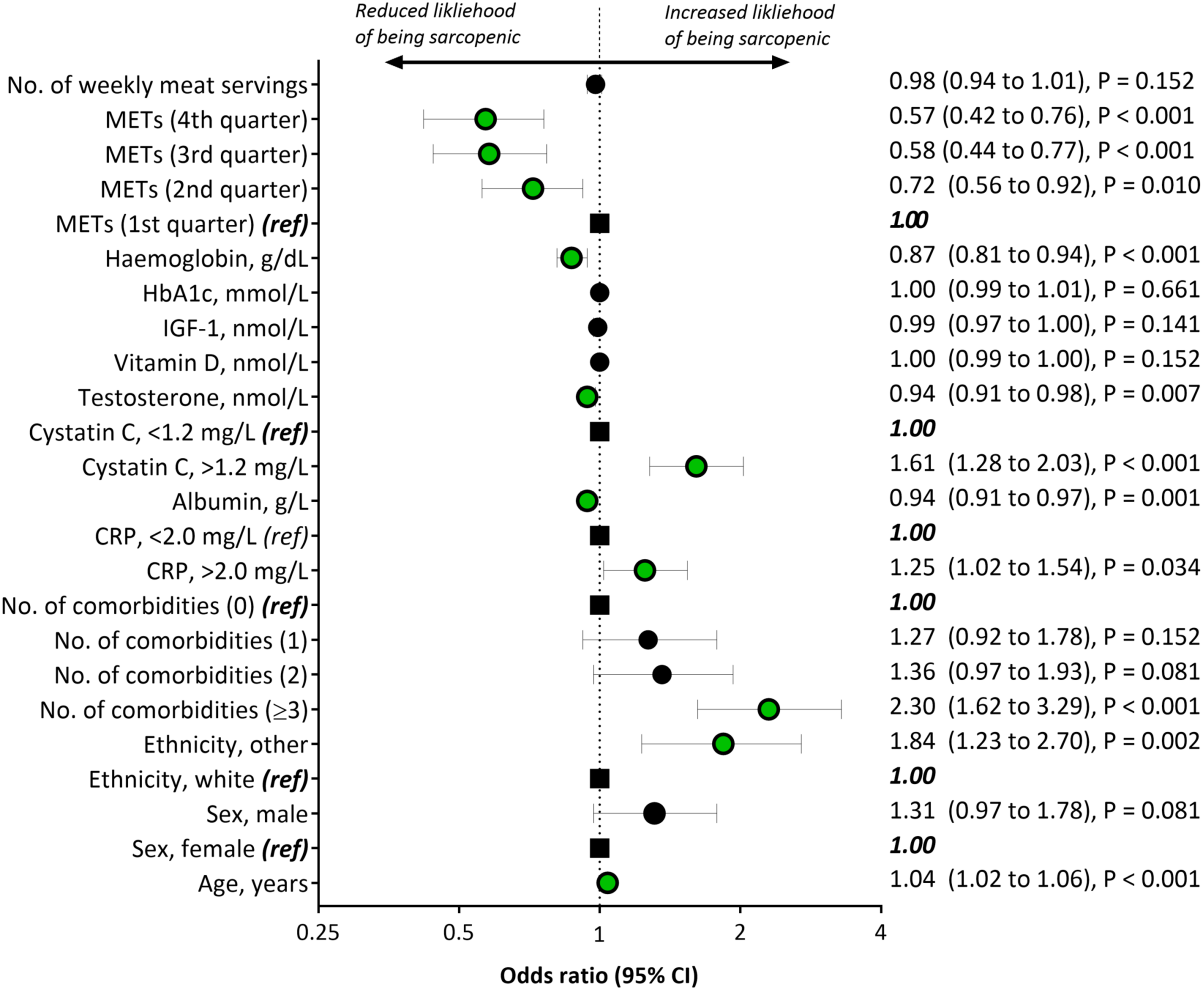
Associations of risk factors with probable sarcopenia in participants with chronic kidney disease (CKD). CI, confidence interval; CRP, C‐reactive protein; IGF, insulin‐like growth factor; METs, metabolic equivalent of task (quartiles defined retrospectively for analysis). Black closed circle = non‐significant; green closed circle = significant; large black square = reference (ref).

*Table* 2 shows the differences between probable sarcopenic and non‐sarcopenic participants with CKD. Sarcopenia was significantly associated with being older and of a non‐White ethnicity. Participants with three or more comorbidities were 2.3 times more likely to be sarcopenic [odds ratio (OR) = 2.30, 95% CI: 1.62 to 3.29; *P* < 0.001]. Sarcopenic CKD participants had greater inflammation (CRP) and poorer renal function (cystatin C), as well as lower serum albumin, testosterone, and haemoglobin levels. Sarcopenia was associated with physical inactivity: participants in the highest quartile of METs per week were 43% less likely to have sarcopenia compared to those in the lowest quartile (OR = 0.57, 95% CI: 0.42 to 0.76; *P* < 0.001). Levels of circulating vitamin D, IGF‐1, HbA1c, and the number of weekly meat servings were not associated with sarcopenic status (*Figure* 2).

**Table 2:** Risk factors for probable sarcopenia in individuals with CKD

	Sarcopenic (*n* = 844)	Non‐sarcopenic (*n* = 7890)	*P* value
Age, years	63.9 (4.5)	62.7 (5.9)	<0.001
Sex, male, *n* (%)	364 (43%)	3678 (47%)	0.058
Ethnicity			
White	769 (91%)	7549 (96%)	<0.001
Other	75 (9%)	341 (4%)	
No. of comorbidities			
0	84 (10%)	1887 (24%)	<0.001
1	238 (28%)	2695 (34%)	
2	227 (27%)	1932 (24%)	
≥3	295 (35%)	1376 (17%)	
Albumin, g/L	43.4 (3.2)	44.5 (2.8)	<0.001
CRP, mg/L^a^	2.9 (1.3–6.2)	1.9 (1.0–3.9)	<0.001
Testosterone, nmol/L^a^	4.3 (1.0–10.0)	6.1 (1.0–10.9)	0.015
HbA1c, mmol/L^a^	41.5 (12.0)	39.0 (8.7)	<0.001
Cystatin C, mg/L	1.5 (0.5)	1.3 (0.4)	<0.001
BMI, kg/m^2^	29.8 (5.8)	29.2 (5.1)	0.012
Vitamin D (nmol/L)	46.8 (23.0)	50.1 (22.1)	<0.001
IGF‐1 (nmol/L)	41.5 (12.0)	39.0 (8.7)	0.008
Haemoglobin, g/dL	13.2 (1.5)	13.9 (1.4)	<0.001
No. of weekly meat servings	7.3 (3.2)	7.5 (2.9)	0.048
METs exercise^a^	74.3 (00.0–433.1)	272.8 (37.1–810.0)	<0.001

BMI, body mass index; CKD, chronic kidney disease; CRP, C‐reactive protein; IGF, insulin‐like growth factor; METs, metabolic equivalent of task.

^a^ Median and interquartile range.

Data presented as mean and standard deviation, unless otherwise indicated.

Missing data for these variables can be found in Table S8.

### Sarcopenia and risk of all‐cause mortality

**Figure 3:**
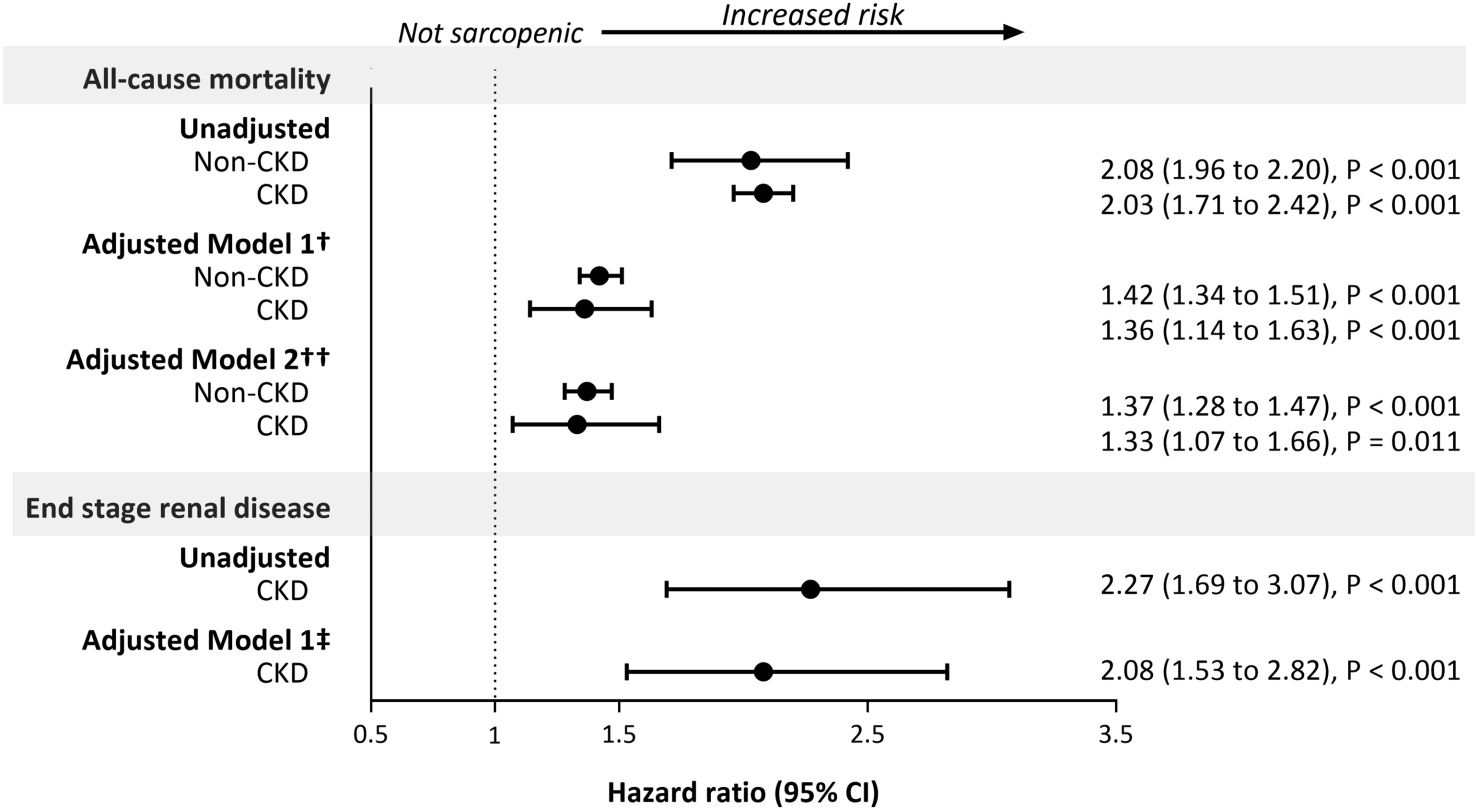
Hazard ratios of all‐cause mortality and risk of end‐stage renal disease for probable sarcopenia, by chronic kidney disease status. Hazard ratios comparing ‘probable sarcopenia’ vs no sarcopenia status. ^†^Model 1 adjusted for non‐modifiable risk factors: age, ethnicity, sex, number of comorbidities; ^††^Model 2 adjusted for the variables in Model 1 plus modifiable risk factors: C‐reactive protein, albumin, body mass index, testosterone, and haemoglobin concentration; ^‡^Adjusted for age, sex, ethnicity, and number of comorbidities.

During 9.0 (interquartile range: 8.3–9.7) years of follow‐up, 152 (18%) deaths occurred in participants with probable sarcopenia and CKD and 1310 (6%) in those sarcopenic without CKD. The hazard ratio of all‐cause mortality was comparable among all participants (both CKD and non‐CKD) with sarcopenia (*Figure* 3 and *Table* S12). Participants with CKD and sarcopenia had a 33% higher relative hazard of mortality compared with CKD individuals without sarcopenia (HR 1.33; 95% CI: 1.07 to 1.66; *P* = 0.011), adjusted for age, sex, ethnicity, number of comorbidities, CRP, albumin, testosterone, Hb, and BMI; in the non‐CKD group, the corresponding HR was 1.37 (1.28 to 1.47; *P* < 0.001). No evidence of an interaction between probable sarcopenia and exposure group was found when fitting the full model with an interaction term (*P* = 0.481).

**Figure 4:**
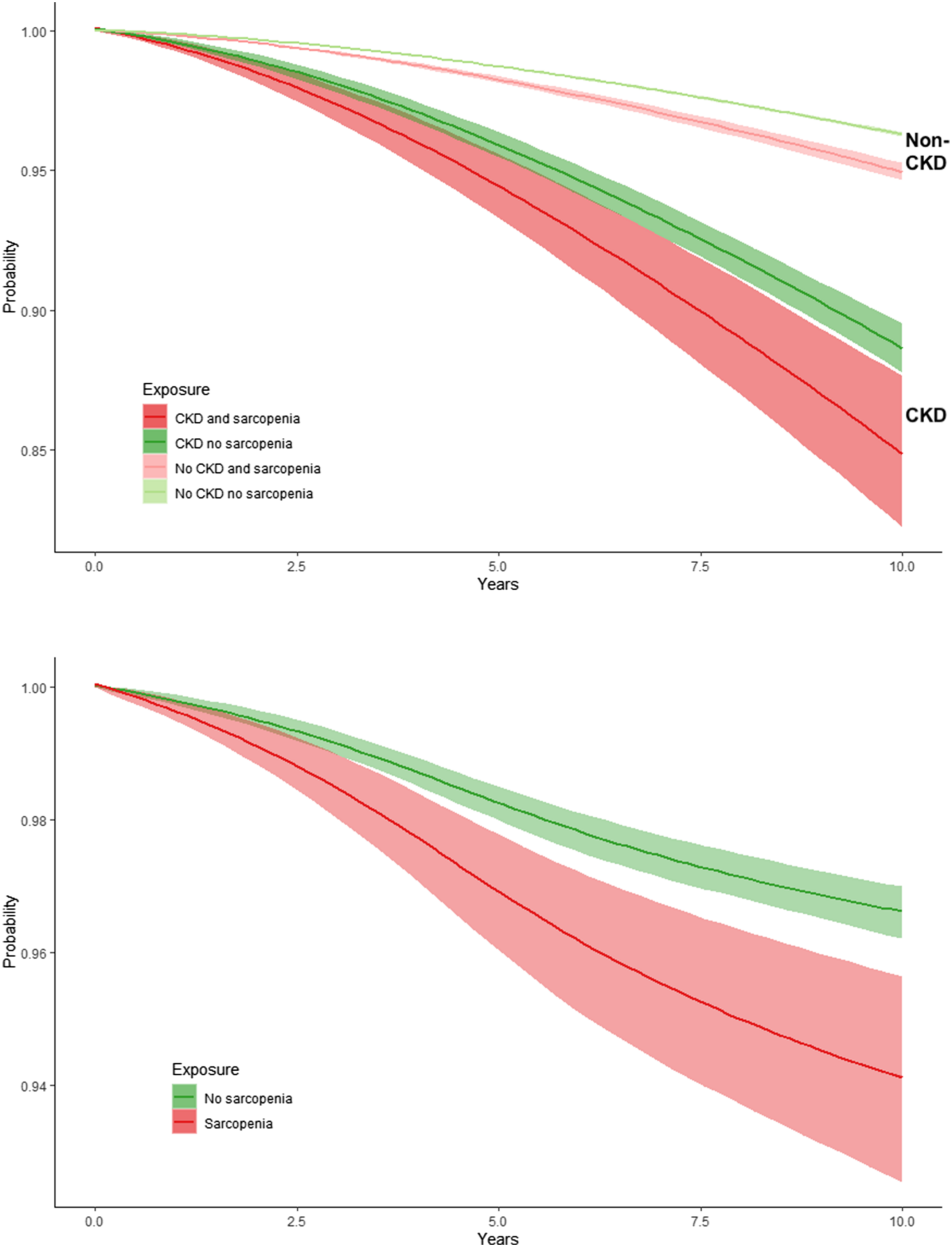
Survival probabilities (with and without developing end‐stage renal disease) in probable sarcopenic and non‐sarcopenic participants, stratified by chronic kidney disease (CKD). Areas indicate 95% confidence intervals.

*Figure* 4 shows the adjusted average survival probability up to 10 years in sarcopenic and non‐sarcopenic participants with and without CKD. In participants with CKD, the 10 years adjusted survival probability was 0.85 (95% CI: 0.82 to 0.88) in those sarcopenic and 0.89 (0.88 to 0.90) in those without sarcopenia, corresponding to a difference of 4% (1% to 7%). In contrast, in non‐CKD participants there was a negligible difference in 10 year survival between those sarcopenic (10 year survival: 0.95; 95% CI: 0.95 to 0.95) and non‐sarcopenic (0.96; 0.96 to 0.96): 1% (1% to 2%).

### Sarcopenia and risk of end‐stage renal disease

In participants with CKD, those with sarcopenia were twice as likely to develop ESRD (HR 2.08 (95% CI: 1.53 to 2.82), *P* < 0.001); adjusted for age, sex, ethnicity, and number of comorbidities) than those without sarcopenia (*Figure* 3 and *Table* S11).

## Discussion

### Summary of findings

We found a probable sarcopenia prevalence of 9.7% among participants with reduced kidney function defined as an eGFR <60 mL/min/1.73 m^2^; this prevalence was approximately double that seen in those without CKD. The prevalence of confirmed and severe sarcopenia in participants with CKD was low, although still twice that observed in those without CKD. Several risk factors for sarcopenia were identified including reduced physical activity, greater inflammation, lower kidney function, and an increased number of comorbidities. Regardless of whether participants had CKD or not, the risk of all‐cause death in those with sarcopenia was approximately one‐third higher. However, when considering the substantially greater baseline mortality risk in the CKD group, the absolute risk of mortality was higher in individuals with CKD, regardless of the presence of sarcopenia in those without CKD. Sarcopenic CKD participants were twice as likely to develop ESRD compared with individuals without sarcopenia.

### Interpretation of findings

In an ageing multimorbid population, sarcopenia is an increasing clinical problem with devastating effects on health and healthcare costs. It is estimated that reducing sarcopenia prevalence by 10% would save approximately $1.1 billion in medical costs per year in the USA.^33^ With the introduction of a sarcopenia ICD‐10 code supporting its growing clinical importance, the lack of uniformity in sarcopenia definitions make its recognition and implementation difficult, especially in those with CKD. No participants in our analysis had a coding of M62.84 for sarcopenia, and only four had an ICD‐10 code of M62.4 (defined as ‘other specified disorders of the muscle’). This is likely due to a lack of sarcopenia recognition and assessment in UK healthcare organizations.^8^ In studies of non‐dialysis CKD, sarcopenia prevalence estimates range widely from 6% to 19%.^12^, ^13^, ^15^, ^20^, ^21^, ^34^, ^35^ However, the ability to compare studies is greatly limited by the diverse definitions employed. The latest criteria by the EWGSOP, as well as the Sarcopenia Definition and Outcomes Consortium (SDOC),^2^ recently supported the use of low muscle strength as the primary parameter of sarcopenia. Termed ‘probable sarcopenia’, its use is intended to allow for simple recognition and better management of sarcopenia, where measurement of muscle mass may not be possible.^4^, ^36^

We found that one in ten participants with CKD had probable sarcopenia, almost twice that observed in those without CKD. Studies investigating low HGS—that is, probable sarcopenia—are limited in non‐dialysis CKD populations. De Souza *et al*.^12^ reported a prevalence of 9% in a sample of 100 elderly CKD patients Stages 2–5; however, the cut‐offs for low HGS were less stringent than those considered in the EWGSOP. Using the same criteria, Zhou *et al*.^34^ observed a sarcopenia prevalence of 29%. In contrast, however, Roshanravan *et al*.^22^ reported that HGS was not impaired in CKD. To our knowledge, only two relatively small Italian studies have utilized the new EWGSOP criteria in a non‐dialysis CKD population. In a sample of 113 elderly (mean age 80 years) patients, Vettoretti *et al*.^23^ found probable sarcopenia was evident in 63% of patients, with confirmed sarcopenia in 24%. Guida *et al*.^21^ found the prevalence of sarcopenia and dynapenia (low HGS) was, respectively, 7.1% and 17.6% in 85 patients.

These values are higher than observed in our cohort of almost 9000 CKD patients where the ‘confirmation’ of sarcopenia through inclusion of low muscle mass resulted in a sarcopenia prevalence of <1%. As such, it is worth asking if the current criteria for sarcopenia is adequate for classifying risk in CKD. Indeed, the inclusion of lean muscle mass in the definition of sarcopenia measured by dual‐energy X‐ray absorptiometry has been questioned.^2^ Cut points from the EWGSOP for ALM/height^2^ are derived from a previous study of 682 young adults from Australia^37^ and potentially highlight limitations in applying these criteria to large international biobank cohorts, but also in populations where greater muscle mass may be observed in the presence of increased adiposity. However, whilst the use of cut‐offs is imperfect for sarcopenia diagnosis,^2^ they do provide a target for appropriate interventions. The low sarcopenia prevalence in our study may also be indicative of the UK Biobank cohort, which has a known ‘healthy responder’ selection bias.^38^ Whilst these factors may explain the low estimates in our analysis, it is important to note that the prevalence of sarcopenia is double that observed in those without CKD regardless of criteria used. Dodds *et al*.^27^ recently investigated sarcopenia across all UK Biobank participants finding a probable sarcopenia prevalence of 5.3%. In participants with a kidney condition, the prevalence was 6.4%. However, these findings are difficult to contrast against ours as health conditions were largely derived from self‐report, whereas we used creatinine‐based eGFR as per international nephrology guidelines.

Whilst the concept of probable sarcopenia is new, the recognition of low muscle strength as a prognostic marker is well established.^39^, ^40^ HGS is shown to be a reliable nutritional marker in dialysis patients^41^ and low HGS predicts mortality in patients with ESRD.^42^, ^43^, ^44^ We found that CKD and non‐CKD participants with probable sarcopenia had a 33% and 37% greater risk of death. However, considering the greater baseline mortality risk in the CKD group, the probability of 10 year survival in sarcopenic CKD patients isapproximately 4% lower compared with non‐sarcopenic CKD patients. We found no meaningful difference in the absolute 10 year mortality risk between sarcopenic and non‐sarcopenic individuals without CKD, suggesting that the presence of reduced kidney function may modify the association between sarcopenia and mortality. Indeed, our findings show that having CKD has a far greater impact on survival than sarcopenia, that is, those without CKD *and* probable sarcopenia had a much better survival that those with CKD and *without* sarcopenia.

In participants with CKD, probable sarcopenia was associated with an increased risk of developing ESRD. Adjustment for age, comorbidities, inflammation, and other factors attenuated the association with mortality but did not fully explain our findings suggesting that sarcopenia is associated with the risk of death independent of these factors. Our work supports research by Chang *et al*.^45^ who described an association between low HGS and an increased risk of mortality and initiation of dialysis in a small study of 128 Taiwanese CKD Stages 1–5 patients. However, in contrast to Chang *et al*. and prior studies in the general population,^39^, ^40^, ^46^ Roshanravan *et al*.^22^ found no association between handgrip and mortality in a cohort of 385 patients CKD Stages 2–4. Several mechanisms may explain the relationship between HGS and outcomes, but low strength is likely an indicator of physical inactivity and other unhealthy behaviours.^39^ In particular, the association of low HGS with inflammation, as well as arterial stiffness and insulin resistance,^45^ may aggravate cardiovascular disease and accelerate kidney function decline.

The underlying complex mechanisms of sarcopenia in the context of CKD are multifactorial. Whilst reductions in muscle strength are likely augmented by changes in muscle mass, the two are not exclusively related, and other detrimenta changes to muscle architecture, such as mitochondrial dysregulation^10^, ^47^ and motor neuron loss, may contribute.^1^ In some individuals, sarcopenia is attributable to ageing (‘primary’ sarcopenia),^1^ and unsurprisingly, we observed that older individuals were more likely to have probable sarcopenia, supporting previous research in CKD.^12^ We observed that participants from a non‐White ethnicity were more likely to be sarcopenic, which is consistent with known ethnic differences in strength and sarcopenic outcomes.^48^

Several studies have reported correlations between sarcopenia and worsening kidney function^49^ and albuminuria.^50^ Using cystatin C, an endogenous filtration marker less influenced by muscle mass compared with creatinine,^51^ we found that sarcopenia was associated with increased serum cystatin C (i.e. decreased kidney function). Low albumin is often used as a biochemical marker of malnutrition and poor nutritional status, which may result in protein synthesis degradation and muscle weakness.^52^ We observed that sarcopenic participants had lower serum albumin, supporting previous research in older adults^52^ and in patients with CKD,^21^ although absolute differences in albumin between sarcopenic and non‐sarcopenia patients were small. We saw no association between with sarcopenia and the weekly meat intake (a crude surrogate for increased dietary protein), although detailed investigation of macronutrients may provide a better understanding of the role of diet in sarcopenia.

Low‐grade inflammation is common in CKD,^11^ and increased levels of inflammatory mediators may contribute to the pathogenesis of sarcopenia.^1^, ^53^ In particular, sarcopenia is strongly associated with elevated serum CRP levels in CKD^17^, ^35^, ^54^ and non‐CKD individuals,^53^ and CRP is likely an indicator of other pro‐inflammatory cytokines that may influence muscle strength through their effect on muscle mass.^55^ However, given the phenotypical complexity of sarcopenia, it is unlikely to be able to be captured by single biological biomarkers. The androgen testosterone is a potent regulator of skeletal muscle mass.^56^ Reduced with ageing, increased testosterone is associated with both better muscle mass and function,^56^ and we found lower serum testosterone in participants with sarcopenia. Nonetheless, whilst modifiable, given the potential unfavourable cardiovascular side‐effect profile, further research is needed into the role of pharmacological testosterone intervention,^1^ and the small absolute differences in testosterone levels between sarcopenic and non‐sarcopenia patients should be noted. Vitamin D deficiency can up‐regulate the ubiquitin–proteasome pathway leading to protein degradation and skeletal muscle atrophy,^11^, ^57^ whilst IGF‐1 is considered a key mediator in the IGF–AkT pathway responsible for maintaining skeletal muscle mass homeostasis.^9^, ^57^ However, we found no association with either vitamin D or IGF‐1 and sarcopenia, which may be due to systemic (i.e. serum), rather than local, measurement of these markers. We observed no association with Hb1Ac, despite known sarcopenia links with dysglycaemia.^18^ However, our CKD group presented with moderately controlled diabetes (a mean Hb1Ac of ~39 mmol/L).

Given the well‐documented effects of physical activity on skeletal muscle synthesis and function, it is unsurprising that physical inactivity was identified as a contributor to sarcopenia. It is well‐recognized that individuals with CKD are extremely inactive,^58^ and although whether lack of physical activity causes sarcopenia or vice‐versa is unclear, the evidence between sarcopenia and physical activity is well‐defined across a wide number of populations including older adults^59^ and patients with CKD.^12^, ^13^ Physical activity, particularly the use of resistance exercise, remains one of the key modifiable risk factors of sarcopenia and is generally considered the ‘primary’ form of treatment.^1^, ^3^

### Methodological considerations

Unexpectedly, the CKD group had a greater absolute ALM than those without CKD. However, as the CKD group exhibited increased body mass and body fat %, when ALM was normalized to BMI, relative muscle mass was reduced compared to the non‐CKD group. It is recognized that increased body mass (and adiposity) may increase muscle size by providing a chronic overload stimulus on the antigravity musculature. However, when muscular strength is normalized to body mass or strength is assessed in other muscles (e.g. forearm muscles as required for HGS), these individuals appear weaker.^60^ Indeed, we found participants with CKD exhibited lower HGS compared with the non‐CKD group. This finding highlights significant shortfalls in the use of ALM (and ALM/height^2^) cut‐offs in the presence of increased adiposity and obesity. We found ALM^BMI^ returned a greater number of sarcopenic participants, and the use of ALM^BMI^ is reportedly a better discriminator of clinically relevant low lean mass in the presence of obesity and poor functional status,^7^ and as such may be the more appropriate criteria in a CKD population. It is also important to note the EWGSOP criteria were developed for the diagnosis of ageing and not CKD‐related sarcopenia. Specific validation studies have not been performed in CKD, and given the difference in potential pathophysiological mechanisms towards sarcopenia, specific diagnostic criteria should be developed for CKD to be used for prognostic purposes.^21^ The finding of increased adiposity in this group also underlines the emerging role of the sarcopenic obesity phenotype in patients with CKD (i.e. low muscle function/mass in the presence of increased fat mass). It is likely that patients exhibiting this phenotype may be at increased risk of adverse health,^20^ and this remains an important topic for future research in this group.

Our analysis is strengthened by the UK Biobank's large prospective cohort of almost a half a million participants, of which approximately 9000 had reduced kidney function. As such, our study is, to our knowledge, the largest investigation into sarcopenia in those with CKD. We were able to classify CKD using creatinine‐derived eGFR, rather than relying on self‐reported status as in other studies.^27^ An important limitation of UK Biobank is its low initial response rate and evidence of a ‘healthy responder’ bias,^38^ which, alongside participants presenting with mild to moderate CKD disease, may explain the low prevalence of sarcopenia in our analysis. It is unlikely that the direction of the association between sarcopenia and outcome is different in respondents and non‐respondents, and hence, representativeness is not a major concern in our analysis, as also empirically demonstrated.^61^ The differences in risk factors (e.g. CRP, albumin, and testosterone) between those with and with sarcopenia should be treated cautiously. Although their effects on sarcopenic development are supported by the literature, the small absolute differences observed lack clinical utility and are likely statistically significant due to the large sample size included.

## Conclusions

This study shows that in individuals with CKD, the prevalence of sarcopenia, regardless of the criteria used, is double that in those without CKD. The pathophysiology of sarcopenia is multifactorial and complex with modifiable and non‐modifiable risk factors. Probable sarcopenia, that is, low muscle strength, is associated with an increased risk of premature mortality and, in individuals with CKD, with an increased risk of ESRD. Sarcopenia is an important and growing clinical problem, even in those with early mild to moderate disease classification; a timely identification of those at risk of sarcopenia may provide prognostic information to healthcare professionals. Based on our findings, we recommend the measurement of sarcopenia should be incorporated into clinical practice and the ICD‐10‐CM diagnosis code M62.84 used for all patients with probable sarcopenia. Appropriate physical activity and exercise remains the primary treatment of sarcopenia.

## Funding

The UK Biobank was supported by the Wellcome Trust, Medical Research Council, Department of Health, Scottish Government, and Northwest Regional Development Agency. It has also had funding from the Welsh Assembly Government and British Heart Foundation. The research was designed, conducted, analysed, and interpreted by the authors entirely independently of the funding sources. No specific funding was awarded for production of this work. UK Biobank access fees were covered by the Health Research Leicester Biomedical Research Centre (BRC). J.M. was part‐funded by the Stoneygate Trust for the duration of this project and is supported by the Leicester Real World Evidence Centre. T.J.W., C.J.L., L.A.B., A.C.S. are part‐funded by the Stoneygate Trust and supported by the National Institute for Health Research Leicester Biomedical Research Centre (BRC). T.Y. is supported by the National Institute for Health Research Leicester Biomedical Research Centre (BRC). E.L.W. was supported by a Kidney Research UK Post‐doctoral Fellowship. F.Z. is supported by the NIHR Applied Research Collaboration (ARC) East Midlands.

## Author contributions

T. J. W. and A. C. S. conceived the idea of the study; A. C. S. and T. J. W. acquired the data; J. M. carried out the statistical analysis; T. J. W., L. A. B., E. L. W., C. J. L., T. Y., F. Z., and A. C. S. interpreted the findings; and T. J. W. drafted the manuscript. J. M. and T. J. W. had full access to all the data in the study and take responsibility for the integrity of the data and the accuracy of the data analysis. T. Y. and F. Z. provided input in the analysis; all authors critically reviewed the manuscript, and T. J. W. revised the manuscript for final submission. All authors gave final approval.

## Data availability statement

Researchers can apply to use the UK Biobank resource and access the data used. No additional data are available.

## Conflict of interests

The author(s) declared no potential conflicts of interest with respect to the research, authorship, and/or publication of this article. The authors declare that they have no competing interests. TY reports grants from NIHR during the conduct of the study.

## Supporting information


**Data S1.** Supporting information
**Table S1.** The Strengthening the Reporting of Observational Studies in Epidemiology (STROBE) guidelines for reporting observational studies checklist.
**Table S2.** List of comorbidities
**Table S3.** S3—Sarcopenia assessment details
**Table S4.** Biomarker assay procedures
**Table S5.** Calculation of physical activity and dietary variables
**Table S5.** Algorithmically defined end‐stage renal disease report
**Table S7.** Mortality data: linkage from national death registries
**Table S8.** Missing data for Table 1 (Participant characteristics)
**Table S9.** Missing data for Table 2 (Risk factors for probable sarcopenia in CKD)
**Table S10.** Missing data for Figure 1 (Prevalence of sarcopenia status in CKD and non‐CKD participants)
**Table S11.** Participant characteristics stratified for sarcopenic status
**Table S12.** Number of events, hazard ratios and 95% confidence intervals of all‐cause mortality and risk of end‐stage renal disease, by sarcopenia status and CKD

## References

[1] Cruz‐Jentoft AJ , Sayer AA . Sarcopenia. Lancet 2019;393:2636–2646. 31171417 10.1016/S0140-6736(19)31138-9

[2] Cesari M , Kuchel GA . Role of sarcopenia definition and diagnosis in clinical care: moving from risk assessment to mechanism‐guided interventions. J Am Geriatr Soc 2020;68:1406–1409. 32633862 10.1111/jgs.16575

[3] Dent E , Morley JE , Cruz‐Jentoft AJ , Arai H , Kritchevsky SB , Guralnik J , et al. International clinical practice guidelines for sarcopenia (ICFSR): screening, diagnosis and management. J Nutr Health Aging 2018;22:1148–1161.3049882010.1007/s12603-018-1139-9

[4] Cruz‐Jentoft AJ , Bahat G , Bauer J , Boirie Y , Bruyère O , Cederholm T , et al. Sarcopenia: revised European consensus on definition and diagnosis. Age Ageing 2019;48:16–31. 30312372 10.1093/ageing/afy169 PMC6322506

[5] Beaudart C , Zaaria M , Pasleau F , Reginster JY , Bruyère O . Health outcomes of sarcopenia: a systematic review and meta‐analysis. PLoS One 2017;12:e0169548. 28095426 10.1371/journal.pone.0169548 PMC5240970

[6] Norman K , Otten L . Financial impact of sarcopenia or low muscle mass—a short review. Clin Nutr 2019;38:1489–1495. 30316536 10.1016/j.clnu.2018.09.026

[7] Dam TT , Peters KW , Fragala M , Cawthon PM , Harris TB , McLean R , et al. An evidence‐based comparison of operational criteria for the presence of sarcopenia. J Gerontol A Biol Sci Med Sci 2014;69: 584–590. 24737561 10.1093/gerona/glu013 PMC3991139

[8] Offord NJ , Clegg A , Turner G , Dodds RM , Sayer AA , Witham MD . Current practice in the diagnosis and management of sarcopenia and frailty—results from a UK‐wide survey. J Frailty Sarcopenia Falls 2019;4:71–77. 32300721 10.22540/JFSF-04-071 PMC7155363

[9] Moorthi RN , Avin KG . Clinical relevance of sarcopenia in chronic kidney disease. Curr Opin Nephrol Hypertens 2017;26:219–228. 28198733 10.1097/MNH.0000000000000318 PMC5860815

[10] Roshanravan B , Gamboa J , Wilund K . Exercise and CKD: skeletal muscle dysfunction and practical application of exercise to prevent and treat physical impairments in CKD. Am J Kidney Dis 2017;69:837–852. 28427790 10.1053/j.ajkd.2017.01.051 PMC5441955

[11] Fahal IH . Uraemic sarcopenia: aetiology and implications. Nephrol Dial Transplant 2014;29:1655–1665. 23625972 10.1093/ndt/gft070

[12] de Souza VA , Oliveira D , Barbosa SR , Corrêa JOA , Colugnati FAB , Mansur HN , et al. Sarcopenia in patients with chronic kidney disease not yet on dialysis: analysis of the prevalence and associated factors. PLoS One 2017;12:e0176230. 28448584 10.1371/journal.pone.0176230 PMC5407780

[13] Hirai K , Ookawara S , Morishita Y . Sarcopenia and physical inactivity in patients with chronic kidney disease. Nephrourol Mon 2016;8:e37443. 27570755 10.5812/numonthly.37443 PMC4983408

[14] Ren H , Gong D , Jia F , Xu B , Liu Z . Sarcopenia in patients undergoing maintenance hemodialysis: incidence rate, risk factors and its effect on survival risk. Ren Fail 2016;38:364–371. 26738817 10.3109/0886022X.2015.1132173

[15] Wilkinson TJ , Nixon DGD , Richler‐Potts D , Neale J , Song Y , Smith AC . Identification of the most clinically useful skeletal muscle mass indices pertinent to sarcopenia and physical performance in chronic kidney disease. Nephrology (Carlton) 2020;25:467–474. 31707760 10.1111/nep.13678

[16] Isoyama N , Qureshi AR , Avesani CM , Lindholm B , Barany P , Heimburger O , et al. Comparative associations of muscle mass and muscle strength with mortality in dialysis patients. Clin J Am Soc Nephrol 2014;9:1720–1728. 25074839 10.2215/CJN.10261013 PMC4186520

[17] Kim JK , Choi SR , Choi MJ , Kim SG , Lee YK , Noh JW , et al. Prevalence of and factors associated with sarcopenia in elderly patients with end‐stage renal disease. Clin Nutr 2014;33:64–68. 23631844 10.1016/j.clnu.2013.04.002

[18] Mori K , Nishide K , Okuno S , Shoji T , Emoto M , Tsduda A , et al. Impact of diabetes on sarcopenia and mortality in patients undergoing hemodialysis. BMC Nephrol 2019;20:105. 30922266 10.1186/s12882-019-1271-8 PMC6437886

[19] Kim JK , Kim SG , Oh JE , Lee YK , Noh JW , Kim HJ , et al. Impact of sarcopenia on long‐term mortality and cardiovascular events in patients undergoing hemodialysis. Korean J Intern Med 2019;34:599–607. 29161801 10.3904/kjim.2017.083 PMC6506738

[20] Androga L , Sharma D , Amodu A , Abramowitz MK . Sarcopenia, obesity, and mortality in US adults with and without chronic kidney disease. Kidney Int Rep 2017;2:201–211. 28439567 10.1016/j.ekir.2016.10.008 PMC5399775

[21] Guida B , Maro MD , Lauro MD , Lauro TD , Trio R , Santillo M , et al. Identification of sarcopenia and dynapenia in CKD predialysis patients with EGWSOP2 criteria: an observational, cross‐sectional study. Nutrition 2020;78:110815. 32480255 10.1016/j.nut.2020.110815

[22] Roshanravan B , Robinson‐Cohen C , Patel KV , Ayers E , Littman AJ , Boer IHD , et al. Association between physical performance and all‐cause mortality in CKD. J Am Soc Nephrol 2013;24:822–830. 23599380 10.1681/ASN.2012070702 PMC3636794

[23] Vettoretti S , Caldiroli L , Armelloni S , Ferrari C , Cesari M , Messa P . Sarcopenia is associated with malnutrition but not with systemic inflammation in older persons with advanced CKD. Nutrients 2019;11:1378. 10.3390/nu11061378 PMC6628018 31248132

[24] Allen N , Sudlow C , Downey P , Peakman T , Danesh J , Elliott P , et al. UK Biobank: current status and what it means for epidemiology. Health Policy Tech 2012;1:123–126.

[25] Levey AS , Eckardt KU , Tsukamoto Y , Levin A , Coresh J , Rossert J , et al. Definition and classification of chronic kidney disease: a position statement from Kidney Disease: Improving Global Outcomes (KDIGO). Kidney Int 2005;67:2089–2100. 15882252 10.1111/j.1523-1755.2005.00365.x

[26] Chudasama YV , Khunti KK , Zaccardi F , Rowlands AV , Yates T , Gillies CL , et al. Physical activity, multimorbidity, and life expectancy: a UK Biobank longitudinal study. BMC Med 2019;17:108. 31186007 10.1186/s12916-019-1339-0 PMC6560907

[27] Dodds RM , Granic A , Robinson SM , Sayer AA . Sarcopenia, long‐term conditions, and multimorbidity: findings from UK Biobank participants. J Cachexia Sarcopenia Muscle 2020;11:62–68. 31886632 10.1002/jcsm.12503 PMC7015236

[28] Studenski SA , Peters KW , Alley DE , Cawthon PM , McLean RR , Harris TB , et al. The FNIH sarcopenia project: rationale, study description, conference recommendations, and final estimates. J Gerontol A Biol Sci Med Sci 2014;69:547–558. 24737557 10.1093/gerona/glu010 PMC3991146

[29] Syddall HE , Westbury LD , Cooper C , Sayer AA . Self‐reported walking speed: a useful marker of physical performance among community‐dwelling older people? J Am Med Dir Assoc 2015;16:323–328. 25523286 10.1016/j.jamda.2014.11.004 PMC6600869

[30] Bradbury KE , Young HJ , Guo W , Key TJ . Dietary assessment in UK Biobank: an evaluation of the performance of the touchscreen dietary questionnaire. J Nutr Sci 2018;7:e6. 29430297 10.1017/jns.2017.66 PMC5799609

[31] Herrington WG , Smith M , Bankhead C , Matsushita K , Stevens S , Holt T , et al. Body‐mass index and risk of advanced chronic kidney disease: prospective analyses from a primary care cohort of 1.4 million adults in England. PLoS One 2017;12:e0173515. 28273171 10.1371/journal.pone.0173515 PMC5342319

[32] Royston P , Parmar MK . Flexible parametric proportional‐hazards and proportional‐odds models for censored survival data, with application to prognostic modelling and estimation of treatment effects. Stat Med 2002;21:2175–2197. 12210632 10.1002/sim.1203

[33] Janssen I , Shepard DS , Katzmarzyk PT , Roubenoff R . The healthcare costs of sarcopenia in the United States. J Am Geriatr Soc 2004;52:80–85. 14687319 10.1111/j.1532-5415.2004.52014.x

[34] Zhou Y , Hellberg M , Svensson P , Höglund P , Clyne N . Sarcopenia and relationships between muscle mass, measured glomerular filtration rate and physical function in patients with chronic kidney disease stages 3–5. Nephrol Dial Transplant 2018;33:342–348. 28340152 10.1093/ndt/gfw466

[35] Pereira RA , Cordeiro AC , Avesani CM , Carrero JJ , Lindholm B , Amoaro FC , et al. Sarcopenia in chronic kidney disease on conservative therapy: prevalence and association with mortality. Nephrol Dial Transplant 2015;30:1718–1725. 25999376 10.1093/ndt/gfv133

[36] Dodds RM , Murray JC , Robinson SM , Sayer AA . The identification of probable sarcopenia in early old age based on the SARC‐F tool and clinical suspicion: findings from the 1946 British birth cohort. Eur Geriatr Med 2020;11:433–441. 32297269 10.1007/s41999-020-00310-5 PMC7280335

[37] Gould H , Brennan SL , Kotowicz MA , Nicholson GC , Pasco JA . Total and appendicular lean mass reference ranges for Australian men and women: the Geelong osteoporosis study. Calcif Tissue Int 2014;94:363–372. 24390582 10.1007/s00223-013-9830-7

[38] Fry A , Littlejohns TJ , Sudlow C , Doherty N , Adamska L , Sposen T , et al. Comparison of sociodemographic and health‐related characteristics of UK Biobank participants with those of the general population. Am J Epidemiol 2017;186:1026–1034. 28641372 10.1093/aje/kwx246 PMC5860371

[39] Soysal P , Kocyigit SE , Dokuzlar O , Ates Bulut E , Smith L , Isik AT . Relationship between sarcopenia and orthostatic hypotension. Age Ageing 2020;49:959–965. 32614946 10.1093/ageing/afaa077

[40] Celis‐Morales CA , Welsh P , Lyall DM , Steell L , Petermann F , Anderson J , et al. Associations of grip strength with cardiovascular, respiratory, and cancer outcomes and all cause mortality: prospective cohort study of half a million UK Biobank participants. BMJ 2018;361:k1651. 29739772 10.1136/bmj.k1651 PMC5939721

[41] Wang AY , Sea MM , Ho ZS , Lui SF , Li PK , Woo J . Evaluation of handgrip strength as a nutritional marker and prognostic indicator in peritoneal dialysis patients. Am J Clin Nutr 2005;81:79–86. 15640464 10.1093/ajcn/81.1.79

[42] Stenvinkel P , Barany P , Chung SH , Lindholm B , Heimbürger O . A comparative analysis of nutritional parameters as predictors of outcome in male and female ESRD patients. Nephrol Dial Transplant 2002;17:1266–1274. 12105251 10.1093/ndt/17.7.1266

[43] Leal VO , Mafra D , Fouque D , Anjos LA . Use of handgrip strength in the assessment of the muscle function of chronic kidney disease patients on dialysis: a systematic review. Nephrol Dial Transplant 2011;26:1354–1360. 20709742 10.1093/ndt/gfq487

[44] Lee YH , Kim JS , Jung SW , Hwang HS , Moon JY , Jeong KH , et al. Gait speed and handgrip strength as predictors of all‐cause mortality and cardiovascular events in hemodialysis patients. BMC Nephrol 2020;21:166. 32375664 10.1186/s12882-020-01831-8 PMC7203881

[45] Chang YT , Wu HL , Guo HR , Tseng CC , Wang MC , Lin CY , et al. Handgrip strength is an independent predictor of renal outcomes in patients with chronic kidney diseases. Nephrol Dial Transplant 2011;26:3588–3595. 21444362 10.1093/ndt/gfr013

[46] Zaccardi F , Franks PW , Dudbridge F , Davies MJ , Khunti K , Yates T . Mortality risk comparing walking pace to handgrip strength and a healthy lifestyle: a UK Biobank study. Eur J Prev Cardiol 2019;2019:2047487319885041. 10.1177/2047487319885041 34247229

[47] Watson EL , Baker LA , Wilkinson TJ , Gould DW , Graham‐Brown MPM , Major RW , et al. Reductions in skeletal muscle mitochondrial mass are not restored following exercise training in patients with chronic kidney disease. FASEB J 2020;34:1755–1767. 31914685 10.1096/fj.201901936RR

[48] Silva AM , Shen W , Heo M , Gallagher D , Wang Z , Sardinha LB , et al. Ethnicity‐related skeletal muscle differences across the lifespan. Am J Hum Biol 2010;22:76–82. 19533617 10.1002/ajhb.20956 PMC2795070

[49] Foley RN , Wang C , Ishani A , Collins AJ , Murray AM . Kidney function and sarcopenia in the United States general population: NHANES III. Am J Nephrol 2007;27:279–286. 17440263 10.1159/000101827

[50] Han E , Lee YH , Kim G , Lee BW , Kang ES , Ahn CW , et al. Sarcopenia is associated with albuminuria independently of hypertension and diabetes: KNHANES 2008‐2011. Metabolism 2016;65:1531–1540. 27621188 10.1016/j.metabol.2016.07.003

[51] Wilkinson TJ , Gould DW , Watson EL , Smith AC . Commentary: Renal function estimation and Cockcroft‐Gault formulas for predicting cardiovascular mortality in population‐based, cardiovascular risk, heart failure and post‐myocardial infarction cohorts: the Heart ‘OMics’ in AGEing (HOMAGE) and the high‐risk myocardial infarction database initiatives. Front Med (Lausanne) 2017;4:77. 28660190 10.3389/fmed.2017.00077 PMC5467000

[52] Uemura K , Doi T , Lee S , Shimada H . Sarcopenia and low serum albumin level synergistically increase the risk of incident disability in older adults. J Am Med Dir Assoc 2019;20:90–93. 30056011 10.1016/j.jamda.2018.06.011

[53] Bano G , Trevisan C , Carraro S , Solmi M , Luchini C , Stubbs B , et al. Inflammation and sarcopenia: a systematic review and meta‐analysis. Maturitas 2017;96:10–15. 28041587 10.1016/j.maturitas.2016.11.006

[54] Kaizu Y , Ohkawa S , Odamaki M , Ikegaya N , Hibi I , Miyaji K , et al. Association between inflammatory mediators and muscle mass in long‐term hemodialysis patients. Am J Kidney Dis 2003;42:295–302. 12900811 10.1016/s0272-6386(03)00654-1

[55] Visser M , Pahor M , Taaffe DR , Goodpaster BH , Simonsick EM , Newman AB , et al. Relationship of interleukin‐6 and tumor necrosis factor‐alpha with muscle mass and muscle strength in elderly men and women: the Health ABC Study. J Gerontol A Biol Sci Med Sci 2002;57:M326–M332. 11983728 10.1093/gerona/57.5.m326

[56] Shin MJ , Jeon YK , Kim IJ . Testosterone and sarcopenia. World J Mens Health 2018;36:192–198. 29756416 10.5534/wjmh.180001 PMC6119844

[57] Molina P , Carrero JJ , Bover J , Chauveau P , Mazzaferro S , Torres PU , et al. Vitamin D, a modulator of musculoskeletal health in chronic kidney disease. J Cachexia Sarcopenia Muscle 2017;8:686–701. 28675610 10.1002/jcsm.12218 PMC5659055

[58] Wilkinson TJ , Clarke AL , Nixon DGD , Hull KL , Song Y , Burton JO , et al. Prevalence and correlates of physical activity across kidney disease stages: an observational multicentre study. Nephrol Dial Transplant 2019;36:641–649. 10.1093/ndt/gfz235 31725147

[59] Steffl M , Bohannon RW , Sontakova L , Tufano JJ , Shiells K , Holmerova I . Relationship between sarcopenia and physical activity in older people: a systematic review and meta‐analysis. Clin Interv Aging 2017;12:835–845. 28553092 10.2147/CIA.S132940 PMC5441519

[60] Tomlinson DJ , Erskine RM , Morse CI , Winwood K , Onambélé‐Pearson G . The impact of obesity on skeletal muscle strength and structure through adolescence to old age. Biogerontology 2016;17:467–483. 26667010 10.1007/s10522-015-9626-4 PMC4889641

[61] Batty GD , Gale CR , Kivimäki M , Deary IJ , Bell S . Comparison of risk factor associations in UK Biobank against representative, general population based studies with conventional response rates: prospective cohort study and individual participant meta‐analysis. BMJ 2020;368:m131. 32051121 10.1136/bmj.m131 PMC7190071

[62] von Haehling S , Morley JE , Coats AJS , Anker SD . Ethical guidelines for publishing in the Journal of Cachexia, Sarcopenia and Muscle: update 2019. J Cachexia Sarcopenia Muscle 2019;10:1143–1145. 31661195 10.1002/jcsm.12501 PMC6818444

