# Supplementary material for: Association of sarcopenia with mortality and end‐stage renal disease in those with chronic kidney disease: a UK Biobank study
Source: J Cachexia Sarcopenia Muscle. 2021 May 5;12(3):586–98. doi: 10.1002/jcsm.12705 (PMC8200422; doi:10.1002/jcsm.12705)
Supplement: Supplementary file 1 — Data S1. Supporting information Table S1. The Strengthening the Reporting of Observational Studies in Epidemiology (STROBE) guidelines for reporting observational studies checklist. Table S2. List of comorbidities Table S3. S3—Sarcopenia assessment details Table S4. Biomarker assay procedures Table S5. Calculation of physical activity and dietary variables Table S5. Algorithmically defined end‐stage renal disease report Table S7. Mortality data: linkage from national death registries Table S8. Missing data for Table 1 (Participant characteristics) Table S9. Missing data for Table 2 (Risk factors for probable sarcopenia in CKD) Table S10. Missing data for Figure 1 (Prevalence of sarcopenia status in CKD and non‐CKD participants) Table S11. Participant characteristics stratified for sarcopenic status Table S12. Number of events, hazard ratios and 95% confidence intervals of all‐cause mortality and risk of end‐stage renal disease, by sarcopenia status and CKD [file JCSM-12-586-s001.docx]

**Supplementary material**

*Wilkinson et al. Association of sarcopenia with mortality and end-stage renal disease in people with chronic kidney disease: a UK Biobank study in 428,320 individuals*

S1 – The Strengthening the Reporting of Observational Studies in Epidemiology (STROBE) guidelines for reporting observational studies checklist

S2 – List of comorbidities

S3 - Sarcopenia assessment details

S4 – Biomarker assay procedures

S5 – Calculation of physical activity and dietary variables

S6 - Algorithmically-defined end-stage renal disease report

S7 - Mortality data: linkage from national death registries

S8 - Missing data for Table 1 (Participant characteristics)

S9 - Missing data for Table 2 (Risk factors for probable sarcopenia in CKD)

S10 - Missing data for Figure 1 (Prevalence of sarcopenia status in CKD and non-CKD participants)

S11 – Participant characteristics stratified for sarcopenic status

S12 - Number of events, hazard ratios and 95% confidence intervals of all-cause mortality and risk of end-stage renal disease, by sarcopenia status and CKD

**S1 – The Strengthening the Reporting of Observational Studies in Epidemiology (STROBE) guidelines for reporting observational studies checklist**

|  | **Item No** | **Recommendation** | **Page No** |
| --- | --- | --- | --- |
| **Title and abstract** | 1 | (*a*) Indicate the study’s design with a commonly used term in the title or the abstract | 3 |
|  |  | (*b*) Provide in the abstract an informative and balanced summary of what was done and what was found | 3 |
| **Introduction** | | | |
| Background/rationale | 2 | Explain the scientific background and rationale for the investigation being reported | 5-6 |
| Objectives | 3 | State specific objectives, including any prespecified hypotheses | 6 |
| **Methods** | | | |
| Study design | 4 | Present key elements of study design early in the paper | 7 |
| Setting | 5 | Describe the setting, locations, and relevant dates, including periods of recruitment, exposure, follow-up, and data collection | 7 |
| Participants | 6 | (*a*) Give the eligibility criteria, and the sources and methods of selection of participants | 7 |
| Variables | 7 | Clearly define all outcomes, exposures, predictors, potential confounders, and effect modifiers. Give diagnostic criteria, if applicable | 7-10 |
| Data sources/ measurement | 8* | For each variable of interest, give sources of data and details of methods of assessment (measurement). Describe comparability of assessment methods if there is more than one group | 7-10 |
| Bias | 9 | Describe any efforts to address potential sources of bias | 7 |
| Study size | 10 | Explain how the study size was arrived at | N/A |
| Quantitative variables | 11 | Explain how quantitative variables were handled in the analyses. If applicable, describe which groupings were chosen and why | 10-11 |
| Statistical methods | 12 | (*a*) Describe all statistical methods, including those used to control for confounding | 10-11 |
|  |  | (*b*) Describe any methods used to examine subgroups and interactions | 10-11 |
|  |  | (*c*) Explain how missing data were addressed | 10-11 |
|  |  | (*d*) If applicable, describe analytical methods taking account of sampling strategy | 10-11 |
|  |  | (*e*) Describe any sensitivity analyses | 10-11 |
| **Results** | | | |
| Participants | 13* | (a) Report numbers of individuals at each stage of study—eg numbers potentially eligible, examined for eligibility, confirmed eligible, included in the study, completing follow-up, and analysed | 12 |
|  |  | (b) Give reasons for non-participation at each stage | N/A |
|  |  | (c) Consider use of a flow diagram | Fig 1 |
| Descriptive data | 14* | (a) Give characteristics of study participants (eg demographic, clinical, social) and information on exposures and potential confounders | 12 |
|  |  | (b) Indicate number of participants with missing data for each variable of interest | Fig 1, supplementary material |
| Outcome data | 15* | Report numbers of outcome events or summary measures | 12-14 |
| Main results | 16 | (*a*) Give unadjusted estimates and, if applicable, confounder-adjusted estimates and their precision (eg, 95% confidence interval). Make clear which confounders were adjusted for and why they were included | 12-14 |
|  |  | (*b*) Report category boundaries when continuous variables were categorized | 12-14 |
|  |  | (*c*) If relevant, consider translating estimates of relative risk into absolute risk for a meaningful time period | 12-14 |
| Other analyses | 17 | Report other analyses done—eg analyses of subgroups and interactions, and sensitivity analyses | N/A |
| **Discussion** | | | |
| Key results | 18 | Summarise key results with reference to study objectives | 15 |
| Limitations | 19 | Discuss limitations of the study, taking into account sources of potential bias or imprecision. Discuss both direction and magnitude of any potential bias | 20-21 |
| Interpretation | 20 | Give a cautious overall interpretation of results considering objectives, limitations, multiplicity of analyses, results from similar studies, and other relevant evidence | 15-22 |
| Generalisability | 21 | Discuss the generalisability (external validity) of the study results | 20-22 |
| **Other information** | | | |
| Funding | 22 | Give the source of funding and the role of the funders for the present study and, if applicable, for the original study on which the present article is based | 31 |

**S2 – List of comorbidities**

We defined comorbidities as being one of the list of chronic conditions identified by Chudasama et al. [24], excluding CKD as this was one of our exposures. We identified these conditions from the cancer code and non-cancer code illness fields in Biobank [datafields 41270, 40001 and 40002], which were self-reported to a nurse at the baseline visit and coded. We then identified if each participant had one or more codes relating to each chronic condition and scored one point for each chronic condition identified. We then categorised this variable as 0, 1, 2 or 3 or more comorbidities.

**List of comorbidities:**

Hypertension, asthma, cancer, depression, diabetes, angina, migraine, eczema or dermatitis, irritable bowel syndrome, myocardial infarction, osteoporosis, stroke, anxiety or panic attacks, rheumatoid arthritis, glaucoma, epilepsy, vestibular disorder, atrial fibrillation, chronic sinusitis, tuberculosis, meningitis, chronic obstructive pulmonary disease, multiple sclerosis, thyroid problem, Parkinson’s disease, prostate problem, bronchiectasis, anaemia, peripheral vascular disease, heart failure, cirrhosis, hepatitis, schizophrenia, dementia, inflammatory bowel disease.

**S3 - Sarcopenia assessment details**

**Handgrip strength**

Handgrip strength was measured using Jamar J00105 hydraulic handheld dynamometer. This measures grip force isometrically and can be adjusted for hand size in five half-inch increments. The dual-scale readout displays isometric grip force from 0 to 200 pounds (90 kg), with a ‘peak-hold’ needle that remains in place once grip is released. The device was calibrated at the start of each day by an assessment centre staff member. This was done by measuring their own grip strength and observing that the needles rise round the dial together and that the red peak-hold needle remains stable and can be read when grip is released.

For the test, the participant was asked to sit upright in a chair and place their forearms on armrests. With dynamometer handle set to the second incremental slot the participant was asked to hold it first in their right hand. The participant’s elbow of the arm holding the dynamometer was against their side and bent to a 90° angle so that their forearm was pointing forwards with their thumb uppermost. Their wrist was straight so that their hand is either pointing forwards or bent slightly outwards. The staff member supported the dynamometer lightly with one hand and rotated the red peak-hold needle anti-clockwise to zero. They explained to the participant that the adjustable handle of the dynamometer does not move while they are gripping it, but it will measure the strength of their grip. The participant was asked to squeeze the handle of the dynamometer as strongly as they could for ~3 seconds. They were given encouragement while doing so. After 3 seconds the participant was asked to stop, the dynamometer taken from them, and the maximum hand grip strength was read in whole kilogram force units as indicated on the outer aspect of the dial by the red peak-hold needle. This value is recorded. The grip strength measurement is repeated using the left hand, and this value is also entered. The maximum value was recorded.

The full handgrip strength protocol available at:

[*http://biobank.ndph.ox.ac.uk/showcase/showcase/docs/Gripstrength.pdf*](about:blank)

**Body composition**

Muscle mass was assessed using bioelectrical impedance analysis (Tanita BC 418ma Body Fat Analyser). This test was not performed in participants who were:

- Wheelchair-bound
- An amputee
- Unable to grip handles of the Tanita analyser
- Unable to stand
- Not willing to remove their shoes
- Wearing a plaster cast
- Pregnant
- Using a pacemaker

Before stepping onto the machine, the participant was asked to place any valuables from their pockets into the tray provided if not already done so. The participant was asked to place their bare feet on the analyser platform feet markings (ensuring no obstruction by trousers) and to keep their feet still and in contact with the platform. The participant was asked to use their hands to grip the two metal handles firmly with arms hanging loosely by their sides.

Appendicular fat-free mass from the bioelectrical impedance analysis device was transformed into ALM using the following equation:

*ALM (kg) = (0.958 * [Appendicular fat-free mass (kg)]) – (0.166 * G) – 0.308*, with G taking the value 0 if female and 1 if male.

Appendicular fat-free mass consisted of the summed fat-free mass of the right arm and leg plus the left arm and leg. The equation was developed by Dodds et al. [25] using data from UK Biobank participants (n=4,350) who underwent both a dual-energy x-ray absorptiometry (DXA) and bioelectrical impedance assessment.

The UK Biobank DXA protocol can be found online at:

*http://biobank.ndph.ox.ac.uk/showcase/showcase/docs/DXA_explan_doc.pdf*

The full bioelectrical impedance analysis protocol available at:

*http://biobank.ndph.ox.ac.uk/showcase/showcase/docs/body_composition.pdf*

**Physical performance (walking pace)**

The UK Biobank does not contain an objective measure of gait speed performance. As a surrogate marker of poor gait speed, and low performance, we considered participants who self-reported being unable to walk or their own walking pace as ‘slow’. During the touchscreen questionnaire part of the assessment centre visit, participants were asked ‘How would you describe your usual walking pace?’; answers included ‘slow pace’, ‘steady average pace’, ‘brisk pace’, ‘none of the above’, and ‘prefer not to answer’.

**S4 – Biomarker assay procedures**

The following information / procedure details is taken from:

[*http://biobank.ndph.ox.ac.uk/showcase/showcase/docs/biomarker_issues.pdf*](about:blank)

A full list of all biomarkers taken for the UK Biobank can be found:

[*http://www.ukbiobank.ac.uk/wp-content/uploads/2018/11/BCM023_ukb_biomarker_panel_website_v1.0-Aug-2015-edit-2018.pdf*](about:blank)

During recruitment of 500,000 participants into UK Biobank, a series of biological samples were collected. The samples were minimally processed at the assessment centres, with most of the processing conducted at the central laboratory using more efficient and reliable automated systems. The only processing that was done immediately at the assessment centre involved inverting the plasma and serum tubes to mix the preservative/anticoagulant with the blood and then allowing the serum tube to clot at room temp for 30 minutes. All tubes were refrigerated (except for the acid citrate dextrose tube which was held at room temperature) until the end of the day when they were packed (with temperature logging devices) and transported to UK Biobank’s central processing and archiving facility in Stockport. At the central laboratory, all samples were predominantly processed using custom-designed industrial-scale automation systems to generate about 25,000 sample aliquots per day (resulting in 15 million 1.4 ml aliquots for the full cohort). A small proportion of samples were manually aliquoted. The extensive use of automation ensured that all the samples were processed quickly, with an average time of 24 ± 2.5 hours between venepuncture and sample storage.

Multiple immunoassay and clinical chemistry analysers were used to measure the biochemistry markers, details of which are provided in online companion documents for serum ([*https://biobank.ndph.ox.ac.uk/showcase/docs/serum_biochemistry.pdf*](about:blank)) and red blood cells ([*https://biobank.ndph.ox.ac.uk/showcase/refer.cgi?id=2405*](about:blank)), respectively. These analysers were designed according to the internationally recognised standard for testing and calibration laboratories (ISO 17025:2005). During the project, the UK Biobank laboratory was successfully externally audited against the ISO 17025:2005 standard on 27th Dec 2015 for the urine and HbA1c assays, and on 17th Oct 2016 for the serum assays.

The biomarkers used in this study are as follows:

| **Name** | **Type** | **Instrumentation** | **Analysis** | **Supplier** |
| --- | --- | --- | --- | --- |
| C-reactive protein | Serum | AU5800 | Immunoturbidimetric -high sensitivity | Beckman Coulter |
| Cystatin C | Serum | ADVIA 1800 | Latex enhanced  immunoturbidimetric | Siemens |
| Haemoglobin | RBC | LH750 | Haematology analyser | Beckman Coulter |
| Creatinine | Serum | AU5800 | Enzymatic | Beckman Coulter |
| Vitamin D | Serum | LIASON XL | CLIA | DiaSorin Ltd |
| Testosterone | Serum | Unicel DxI 800 | One step competitive | Beckman Coulter |
| IGF-1 | Serum | LIASON XL | CLIA | DiaSorin Ltd. |
| HbA1c | RBC | VARIANT II Turbo | HPLC | Bio-Rad |
| Albumin | Serum | AU5800 | BCG | Beckman Coulter |

**S5 – Calculation of physical activity and dietary variables**

**Metabolic equivalent of task (METs) from exercise**

The approximate METs from exercise were calculated using the fields relating to frequency and duration of pleasure walking, strenuous sport and other exercise. As the duration was a categorical variable each category was assigned a value based on the midpoint of the time interval in minutes, which was then summed for each variable for each person. The frequency for each variable was calculated similarly, with a mid-point of the number of days assigned to each category and then summed. A total weekly duration for each of pleasure walking, strenuous sport and other exercise were calculated by multiplying the corresponding duration and frequency. This was then used to calculate the total METs from exercise by multiplying the total weekly minutes of pleasure walking by 3.3, other exercise by 4 and strenuous sports by 8, and then summing the three variables [Ainswoth et al., 2000; Chudasama et al., 2019]. Participants who were missing information on any of the three variables were regarded as having missing information for the total METs minutes.

**Approximate number of servings of meat or fish**

The approximate total consumption of meat or fish was calculated from the UK Biobank fields: oily fish, non-oily fish, processed meat, poultry, beef, lamb and pork intake. As these were categorical variables a value based on the mid-point of the number of portions consumed per week was calculated for each variable, these were then summed to create the approximate number of servings per week.

**S6 - Algorithmically-defined end-stage renal disease report**

The following information is taken from:

[*http://biobank.ctsu.ox.ac.uk/crystal/crystal/docs/alg_outcome_esrd.pdf*](about:blank)

End-stage renal disease (ESRD) is treated with renal replacement therapy (RRT) which can be readily identified from hospital admission data. However, RRT is also used to treat acute kidney injury (AKI) which epidemiologists may want to study separately. The UK Biobank therefore devised an algorithm that identifies RRT in UK Biobank participants, and then selects the subset of participants with other diagnoses or procedures, which indicate ESRD.

The algorithm detects cases of ESRD using self-report codes or in hospital admissions data by using ICD-10 and OPCS4 codes (these codes can be found in Appendix 1, link below) to identify participants who received any RRT (and within this category those who received a kidney transplant or peritoneal dialysis which was assumed to be for maintenance RRT), and those with indicators of CKD stage 5. Participants who received a kidney transplant or peritoneal dialysis are assumed to be ESRD cases. In order to exclude cases of AKI, the remaining RRT cases are deemed to be ESRD cases only if they have an associated indicator of CKD stage 5 prior within the previous 365 days.

The full process for implementing this algorithm in hospital admissions data is outlined in Appendix 1 found here:

[*http://biobank.ctsu.ox.ac.uk/crystal/crystal/docs/alg_outcome_esrd.pdf*](about:blank)

The principles used by this algorithm have previously been used to successfully identify people with treated ESRD (*Herrington WG, Smith M, Bankhead C, Matsushita K, Stevens S, Holt T, Hobbs FDR, Coresh J, Woodward M. Body-mass Index and Risk of Advanced Chronic Kidney Disease: Prospective Analyses from a Primary Care Cohort of 1.4 Million Adults in England. PloS One 2017 Mar 8;12(3):e0173515*)

**S7 - Mortality data: linkage from national death registries**

The detailed procedure for linkage of UK Biobank participants to mortality data, provided by the NHS Information Centre for participants from England and Wales and by NHS Central Register, Scotland for participants from Scotland, can be found here:

[*http://biobank.ndph.ox.ac.uk/showcase/showcase/docs/DeathLinkage.pdf*](about:blank)

**S8 - Missing data for Table 1 (Participant characteristics)**

|  | **CKD**  **(n=8,767)** |  | **Non-CKD**  **(n=419,553)** |
| --- | --- | --- | --- |
|  |  |  |  |
| Age, years | 0 (0%) |  | 0 (0%) |
| Sex, male, n (%) | 0 (0%) |  | 0 (0%) |
| Ethnicity | 0 (0%) |  | 0 (0%) |
| No. of comorbidities | 0 (0%) |  | 0 (0%) |
| Albumin, g/L | 839 (10%) |  | 35,234 (1%) |
| eGFR, ml/min/1.72m^2^ | 0 (0%) |  | 0 (0%) |
| CRP, mg/L | 30 (0%) |  | 997 (0%) |
| Haemoglobin, g/dL | 194 (2%) |  | 10,663 (0%) |
| Testosterone, nmol/L | 1,130 (13%) |  | 38,181 (1%) |
| HbA1c, mmol/L | 468 (5%) |  | 21,539 (1%) |
| Body mass, kg | 307 (4%) |  | 7,145 (0%) |
| BMI, kg/m^2^ | 307 (4%) |  | 7,152 (0%) |
| Body fat, % |  |  |  |
| Males | 169 (2%) |  | 3,801 (0%) |
| Females | 144 (2%) |  | 3, 582 (0%) |
| ALM, kg |  |  |  |
| Males | 164 (2%) |  | 3,669 (0%) |
| Females | 146 (2%) |  | 3,686 (0%) |
| ALM/height^2^ |  |  |  |
| Males | 168 (2%) |  | 3,833 (0%) |
| Females | 147 (2%) |  | 3,733 (0%) |
| ALM^BMI^ |  |  |  |
| Males | 164 (2%) |  | 3,670 (0%) |
| Females | 146 (2%) |  | 3,686 (0%) |
| Handgrip strength, kg |  |  |  |
| Males | 13 (0%) |  | 471 (0%) |
| Females | 20 (0%) |  | 598 (0%) |
| Slow walking speed, n (%) |  |  |  |
| Males | 71 (1%) |  | 1,374 (0%) |
| Females | 77 (1%) |  | 1,395 (0%) |
|  |  |  |  |

CKD = Chronic kidney disease; eGFR = Estimated glomerular filtration rate; CRP = C-reactive protein; BMI = Body mass index; ALM = Appendicular lean mass

**S9 - Missing data for Table 2 (Risk factors for probable sarcopenia in CKD)**

|  | **Sarcopenic**  **(n=844)** | **Non-sarcopenic**  **(n=7,890)** |
| --- | --- | --- |
|  |  |  |
| Age, years | 0 (0%) | 0 (0%) |
| Sex, male, n (%) | 0 (0%) | 0 (0%) |
| Ethnicity | 0 (0%) | 0 (0%) |
| No. of comorbidities | 0 (0%) | 0 (0%) |
| Albumin, g/L | 91 (11%) | 746 (9%) |
| CRP, mg/L | 5 (1%) | 25 (0%) |
| Testosterone, nmol/L | 143 (17%) | 985 (12%) |
| HbA1c, mmol/L | 43 (5%) | 419 (5%) |
| Cystatin C, mg/L | 0 (0%) | 5 (0%) |
| BMI, kg/m^2^ | 59 (7%) | 231 (3%) |
| Vitamin D (nmol/L) | 21 (2%) | 331 (4%) |
| IGF-1 (nmol/L) | 3 (0%) | 53 (1%) |
| Haemoglobin, g/dL | 21 (2%) | 173 (2%) |
| No. of weekly meat servings | 45 (5%) | 181 (2%) |
| METs exercise | 11 (1%) | 1,510 (19%) |
| METs activity | 658 (78%) | 4,868 (62%) |
|  |  |  |

CKD = Chronic kidney disease; CRP = C-reactive protein; BMI = Body mass index; IGF = Insulin-like growth factor; METs = Metabolic equivalent

**S10 - Missing data for Figure 1 (Prevalence of sarcopenia status in CKD and non-CKD participants)**

|  | **CKD**  **(n=8,767)** | **Non-CKD**  **(n=419,553)** |
| --- | --- | --- |
|  |  |  |
| Probable sarcopenia | 33 (0%) | 1,069 (0%) |
| Confirmed sarcopenia ^a^ | 331 (4%) | 7,983 (2%) |
| Confirmed sarcopenia ^b^ | 326 (4%) | 7,775 (2%) |
| Severe sarcopenia ^a^ | 443 (5%) | 10,202 (2%) |
| Severe sarcopenia ^b^ | 439 (5%) | 10,008 (2%) |
|  |  |  |

^a^ = low muscle mass defined as per EWGSOP (appendicular lean mass adjusted for height^2^);

^b^ = low muscle mass defined as per FNIHSP (appendicular lean mass adjusted for body mass index)

**Table S11 – Participant characteristics stratified for sarcopenic status**

|  | **CKD**  **(n=8,767)** | | **Non-CKD**  **(n=419,553)** | |
| --- | --- | --- | --- | --- |
|  | **Sarcopenic** | **Non-sarcopenic** | **Sarcopenic** | **Non-sarcopenic** |
|  |  |  |  |  |
| Age, years | 63.9 (4.9) | 62.7 (5.9) | 59.5 (7.3) | 55.9 (8.1) |
| Sex, male, n (%) | 364 (43%) | 3,678 (47%) | 8,325 (40%) | 186,774 (47%) |
| Ethnicity |  |  |  |  |
| White | 769 (91%) | 7549 (96%) | 18,594 (89%) | 376,768 (95%) |
| Other | 75 (9%) | 341 (4%) | 2,329 (11%) | 20,793 (5%) |
| No. of comorbidities |  |  |  |  |
| 0 | 84 (10%) | 1,887 (24%) | 6,445 (31%) | 188,803 (47%) |
| 1 | 238 (28%) | 2,695 (34%) | 7,072 (34%) | 132,290 (33%) |
| 2 | 227 (27%) | 1,932 (24%) | 4,277 (20%) | 53,649 (13%) |
| ≥3 | 295 (35%) | 1,376 (17%) | 3,129 (15%) | 22,819 (6%) |
| Albumin, g/L | 43.4 (3.2) | 44.5 (2.8) | 44.6 (2.8) | 45.3 (2.6) |
| eGFR, ml/min/1.72m^2 †^ | 52.1 (45.7-56.8) | 54.7 (49.4-57.8) | 92.8 (83.2-99.3) | 93.4 (84.1-100.6) |
| Stage 3a, n (%) | 648 (77%) | 6,748 (86%) | - | - |
| Stage 3b, n (%) | 149 (18%) | 925 (12%) | - | - |
| Stage 4, n (%) | 41 (5%) | 196 (2%) | - | - |
| CRP, mg/L ^†^ | 2.9 (1.3-6.2) | 1.9 (1.0-3.9) | 1.9 (0.9-4.0) | 1.3 (0.6-2.6) |
| Haemoglobin, g/dL | 13.2 (1.5) | 13.7 (1.4) | 14.0 (1.3) | 14.2 (1.2) |
| Testosterone, nmol/L ^†^ | 4.3 (1.0-10.0) | 6.1 (1.0-10.9) | 1.9 (0.9-10.8) | 5.9 (1.0-11.8) |
| HbA1c, mmol/L | 41.5 (12.0) | 39.0 (8.7) | 37.8 (8.5) | 35.9 (6.6) |
| Body mass, kg | 80.4 (17.6) | 82.2 (16.2) | 75.6 (16.2) | 78.2 (15.8) |
| BMI, kg/m^2^ | 29.8 (5.8) | 29.2 (5.1) | 28.1 (5.3) | 27.4 (4.7) |
| Body fat, % |  |  |  |  |
| Males | 28.8 (6.6) | 27.1 (5.7) | 26.8 (6.3) | 25.1 (5.8) |
| Females | 39.8 (7.0) | 39.0 (6.7) | 38.3 (6.8) | 36.4 (6.9) |
| ALM, kg |  |  |  |  |
| Males | 26.3 (4.8) | 27.2 (4.1) | 25.3 (4.2) | 27.1 (3.9) |
| Females | 18.7 (3.0) | 19.1 (2.7) | 17.8 (2.6) | 18.4 (2.4) |
| ALM/height^2^ |  |  |  |  |
| Males | 9.0 (1.5) | 8.9 (1.2) | 8.6 (1.2) | 8.6 (1.1) |
| Females | 7.3 (1.1) | 7.3 (1.0) | 7.0 (0.9) | 7.0 (0.8) |
| ALM^BMI^ |  |  |  |  |
| Males | 0.9 (0.1) | 0.9 (0.1) | 0.9 (0.1) | 1.0 (0.1) |
| Females | 0.6 (0.1) | 0.7 (0.1) | 0.6 (0.1) | 0.7 (0.1) |
| Handgrip strength, kg |  |  |  |  |
| Males | 21.7 (4.6) | 40.5 (7.8) | 22.0 (4.4) | 42.8 (8.1) |
| Females | 11.5 (3.2) | 24.8 (5.4) | 11.8 (3.0) | 26.0 (5.6) |
| Slow walking speed, n (%) |  |  |  |  |
| Males | 167 (48%) | 672 (19%) | 2,030 (25%) | 12,161 (7%) |
| Females | 200 (44%) | 674 (16%) | 3,151 (26%) | 13,785 (7%) |
|  |  |  |  |  |

Data presented as mean and standard deviation, unless otherwise indicated.

CKD = Chronic kidney disease; eGFR = Estimated glomerular filtration rate; CRP = C-reactive protein; BMI = Body mass index; ALM = Appendicular lean mass

^†^ = median and IQR

**S12 - Number of events, hazard ratios and 95% confidence intervals of all-cause mortality and risk of end-stage renal disease, by sarcopenia status and CKD**

|  | **No. of events** | **Unadjusted HR (95% CI)** | **P value** |
| --- | --- | --- | --- |
|  |  |  |  |
| **All-cause mortality** |  |  |  |
| Probable sarcopenia |  |  |  |
| Non-CKD | 1310 (6%) | 2.08 (1.96 to 2.20) | <0.001 |
| CKD | 152 (18%) | 2.03 (1.71 to 2.42) | <0.001 |
| Confirmed sarcopenia ^a^ |  |  |  |
| Non-CKD | 106 (16%) | 5.42 (4.48 to 6.57) | <0.001 |
| CKD | 9 (32%) | 4.13 (2.14 to 7.96) | <0.001 |
| Confirmed sarcopenia ^b^ |  |  |  |
| Non-CKD | 105 (9%) | 2.84 (2.34 to 3.44) | <0.001 |
| CKD | 17 (26%) | 2.96 (1.83 to 4.79) | <0.001 |
| Severe sarcopenia ^a^ |  |  |  |
| Non-CKD | 42 (29%) | 11.30 (8.35 to 15.30) | <0.001 |
| CKD | 5 (36%) | 5.09 (2.11 to 12.30) | <0.001 |
| Severe sarcopenia ^b^ |  |  |  |
| Non-CKD | 52 (11%) | 3.46 (2.64 to 4.55) | <0.001 |
| CKD | 13 (32%) | 3.99 (2.31 to 6.91) | <0.001 |
|  |  |  |  |
| **End-stage renal disease** |  |  |  |
| Probable sarcopenia | 53 (6%) | 2.27 (1.69 to 3.07) | <0.001 |
| Confirmed sarcopenia ^a^ | 1 (4%) | ^c^ |  |
| Confirmed sarcopenia ^b^ | 6 (9%) | 3.12 (1.39 to 7.01) | 0.006 |
| Severe sarcopenia ^a^ | 0 (0%) | ^c^ |  |
| Severe sarcopenia ^b^ | 4 (10%) | 3.47 (1.29 to 9.31) | 0.014 |
|  |  |  |  |

CKD = Chronic kidney disease; HR = Hazard ratio; 95% CI = 95% confidence interval

^a^ = low muscle mass defined as per EWGSOP (appendicular lean mass adjusted for height^2^);

^b^ = low muscle mass defined as per FNIHSP (appendicular lean mass adjusted for body mass index);

^c^ = too few events for analysis
